# Psychosocial Care after Cancer Diagnosis: Recent Advances and Challenges

**DOI:** 10.3390/cancers14235882

**Published:** 2022-11-29

**Authors:** Laura Tack, Patricia Schofield, Tom Boterberg, Rebecca Chandler, Christopher N. Parris, Philip R. Debruyne

**Affiliations:** 1 Department of Medical Oncology, Kortrijk Cancer Centre, General Hospital Groeninge, 8500 Kortrijk, Belgium; 2 Department of Human Structure and Repair, Ghent University, 9000 Ghent, Belgium; 3 School of Nursing and Midwifery, Faculty of Health, University of Plymouth, Plymouth PL4 8AA, UK; 4 School of Allied Health, Faculty of Health, Education, Medicine and Social Care, Anglia Ruskin University, Chelmsford CM1 1SQ, UK; 5 Medical Technology Research Centre (MTRC), School of Life Sciences, Faculty of Science and Engineering, Anglia Ruskin University, Cambridge CB1 1PT, UK

Psychosocial oncology is coming of age. While the survival rates of cancer patients have increased, many patients suffer from treatment-related long-lasting effects that may adversely affect their mental health and health-related quality of life. Along with the changes in therapeutic strategies, physicians should pay more attention to the psychosocial problems secondary to cancer as it is well recognized that the diagnosis of cancer and its treatment can be extremely stressful and emotional for cancer patients. 

Psychosocial oncology refers to the multidisciplinary subdiscipline of cancer care that was created to improve patients’ mental well-being by offering strategies to help them cope with the demands of treatment and uncertainty of disease outcome in the best possible way. Psychosocial care should be available prior to diagnosis to beyond palliative care and survival. The International Psycho-Oncology Society (IPOS) is the multidisciplinary international body responsible for the psychological, social and behavioural issues in cancer care. They propose an international quality standard, which could have a synergistic effect, in addition to international and national efforts to improve psychosocial cancer care [1]:

1. Psychosocial cancer care should be recognized as a universal human right.
2. Quality cancer care must integrate the psychosocial domain into routine care.
3. Distress should be measured as the sixth vital sign after temperature, blood pressure, pulse, respiratory rate and pain.

That brings us to the topic of this Special Issue: what are the recent advances in psychosocial oncology and what challenges are we facing?

## 1. Psychosocial Consequences of a Cancer Diagnosis

Psychosocial oncology encompasses the management of a myriad of domains such as anxiety [2], fear of cancer recurrence, depression [3], trauma, distress [4], cognitive function, fatigue, sexual function and intimacy, and sleep disturbance [5]. 

Psychological stress or distress played a key role in the EMOTICON trial, where it was hypothesized that there was a causal relationship between distress and the occurrence of subjective or self-reported cancer-related cognitive impairment [6]. Moreover, psychological stress or distress has been known to contribute to the development and progression of cancer [7,8].

In addition, there are many psychosocial aspects of coping with the physical sequelae of cancer and its treatments. Cardiovascular problems, lymphedema, pain, hormonal-related symptoms, infertility, and neuropathy are only a few of the many side effects that may have a psychosocial impact. 

The improved detection of genetic syndromes predisposing patients and their families to cancer also has many psychosocial consequences. Indeed, psychosocial aspects are not limited to cancer patients but also to their caregivers, families, friends, and beyond colleagues and employers. As the impact on patients and their loved ones’ well-being and quality of life is expected to be considerable, it is crucial to examine these psychosocial outcomes to be able to support patients in their coping [9].

Offering an intervention to the patient and extending support and therapy to caregivers and family where possible is the first step towards establishing psychosocial oncology in standard clinical practice. Addressing psychosocial needs and proper management will have a great impact on patient outcomes and quality of life.

Research is focusing on the understanding of different psychosocial aspects, and it is expected that this will lead to improved early detection, advanced effective treatments, and ultimately better prevention, as well as continuously updated international guidelines. Special attention is required for vulnerable patient groups and their caregivers, such as children, older patients, migrant populations, veterans, people with mental disabilities, and adolescents and young adults (AYAs).

## 2. Psychosocial Oncology and Complementary Therapies

Those considered as vulnerable require adequate and often more specialized care. The challenges, that vulnerable patient populations bring requires and contributes to transformations in cancer care. A “one size might not fit all” approach applied in cancer treatment supports holistic care, which implies striving towards physical, psychological, social, and spiritual wellbeing for all cancer patients. Recently, the American Society of Clinical Oncology (ASCO) and the Society of Integrative Oncology (SIO) published clinical guidelines on how best to weave various non-pharmacological pain management strategies into cancer care, as guidance as to when and when not to use these approaches was lacking. The guidelines discuss several mind–body therapies and natural products, with recommendations on acupuncture, reflexology, hypnosis, and massage based on strong evidence [10]. Our research group previously conducted a survey on the acceptability and preferences of patients and caregivers on acupuncture as a complementary therapy for cancer care at the day clinic of the Kortrijk Cancer Centre [11]. Furthermore, within our cancer centre, we installed an art therapy programme taking into account the preferences of the patients and their caregivers [12].

## 3. Accessibility of Psychosocial Oncology

Despite growing recognition that psychosocial care is an essential component of complementary cancer care, psychosocial needs may not be recognized by the clinical team and thus remain unmet [13,14]. This can also be said of caregivers’ needs [14]. This has important implications for patients that may lead to psychosocial morbidity with maladaptive coping, reduced quality of life (QoL), impaired social relationships, suicide risk, longer rehabilitation times, poor treatment adherence and pathological behaviour, familial dysfunction and possibly shorter survival [15]. Counselling patients during their disease trajectory is key to keep patients and their families well-informed and involve them in decision making and, furthermore, to detect the psychological and social needs they might be experiencing [16]. This addresses a great challenge for psychosocial oncology: the creation and implementation of clinical guidelines dedicated to psychosocial interventions within standard clinical practice. Guidelines can educate healthcare professionals on the impact of psychosocial oncology for both the patients and their caregivers, in synergy with medical cancer treatment. In turn, this can also ensure that psychosocial care finds its way to patients who do not receive it to date because of financial, linguistic, cultural and organizational impediments. Furthermore, the set-up of guidelines may highlight gaps in the research evidence.

## 4. Psychosocial Oncology and Telemedicine

Another challenge for the future of healthcare, including psychosocial oncology, is telemedicine. Especially for post-treatment cancer survivorship care, telemedicine services have been increasingly used. Although the efficacy of telemedicine varies by the delivery medium (web-based education programs, online trainings, interactive applications, telephone and online counselling), this novel method appears to have positive effects on psychosocial outcomes [17]. The major advantages of telemedicine comprise the fact that geographical, location and time constraints are no longer encountered by patients and caregivers. Of course, there are still barriers that represent the future challenges for the use of telemedicine in psychosocial oncology, such as the translation to all cancer types, ages, languages and settings. Further, the complexity of incorporating patient-centred care into the design has been identified as one of the barriers to implement telemedicine and may lead to new disparities [17]. Indeed, electronic screening for cancer-related psychosocial distress was found to lead to the underscreening of vulnerable populations who are older in age, non-White and of lower socioeconomic status [18]. Not everyone has easy and direct access to IT media and care should be taken not to exclude patient groups who are less privileged and are often known to be at a higher risk of having psychosocial needs. Facilitators such as the affordability and potential cost savings should promote further research on telemedicine interventions to provide inclusive, equal-opportunity psychosocial care.

To conclude, we are honoured to introduce you to this Special Issue and the many interesting papers that are published herein. They provide new insights on the challenges that psychosocial oncology is facing and report on several new and original research methodologies. We hope to emphasize the importance of psychosocial oncology, with these publications being a source of inspiration to stimulate further research and discussion, as there are diverse and many research directions to be explored for the benefit of patients and their caregivers.
